# Integration of ALV into *CTDSPL* and *CTDSPL2* genes in B-cell lymphomas promotes cell immortalization, migration and survival

**DOI:** 10.18632/oncotarget.19328

**Published:** 2017-07-18

**Authors:** Shelby Winans, Alyssa Flynn, Sanandan Malhotra, Vidya Balagopal, Karen L. Beemon

**Affiliations:** ^1^ Department of Biology, Johns Hopkins University, Baltimore, MD 21218, USA

**Keywords:** CTD small phosphatase like proteins, ALV, lymphoma, immortalization, migration

## Abstract

Avian leukosis virus induces tumors in chickens by integrating into the genome and altering expression of nearby genes. Thus, ALV can be used as an insertional mutagenesis tool to identify novel genes involved in tumorigenesis. Deep sequencing analysis of viral integration sites has identified *CTDSPL* and *CTDSPL2* as common integration sites in ALV-induced B-cell lymphomas, suggesting a potential role in driving oncogenesis. We show that in tumors with integrations in these genes, the viral promoter is driving the expression of a truncated fusion transcript. Overexpression in cultured chick embryo fibroblasts reveals that *CTDSPL* and *CTDSPL2* have oncogenic properties, including promoting cell migration. We also show that *CTDSPL2* has a previously uncharacterized role in protecting cells from apoptosis induced by oxidative stress. Further, the truncated viral fusion transcripts of both *CTDSPL* and *CTDSPL2* promote immortalization in primary cell culture.

## INTRODUCTION

Avian leukosis virus (ALV) is a simple retrovirus that induces tumorigenesis by integrating into the host genome and altering expression of cellular genes [1]. ALV typically induces B-cell lymphomas but has been shown to induce various other neoplasms less frequently [2]. Insertion of the proviral genome into the host cell genome can alter host gene expression in a number of ways. The long terminal repeats (LTRs) contain strong promoter and enhancer elements that can promote nearby gene expression. In addition, the proviral genome can integrate within a gene and disrupt expression or generate truncated protein products with altered functions [3]. ALV integrates relatively randomly into the host genome and thus, is a good tool for insertional mutagenesis screens to identify novel genes involved in cancer [4]. Integrations into a number of proto-oncogenes, including *MYC, MYB, TERT, mir-155, MET* and *EGFR*, have been seen in past screens [1, 5–9].

Our lab has previously identified *CTDSPL* (C-terminal domain small phosphatase-like) and *CTDSPL2* (C-terminal domain small phosphatase-like 2) as common integration sites in ALV-induced B-cell lymphomas [10]. The recurrence and selection of integrations within these genes in tumors suggests that they may be involved in driving tumorigenesis. The CTDSP family of proteins consists of CTDSP1, CTDSP2, CTDSPL and CTDSPL2 proteins, all of which contain a catalytic FCP1 (F-cell production 1) homology domain that functions as a phosphatase [11]. The CTDSP family has been shown to dephosphorylate the C-terminal domain (CTD) of RNA polymerase II *in vitro* [11, 12]. Through this function, this family of proteins is proposed to be important for transcriptional regulation. Most family members preferentially dephosphorylate Ser5 of the CTD and thus control the transition from initiation to processive transcription elongation [11, 12]. CTDSP1, CTDSP2 and CTDSPL have also been shown to play a role in gene silencing, most notably of neuronal gene expression, through interaction with the REST complex [12–14].

The CTDSP proteins are able to act on additional targets as well. For instance, CTDSP1/2/L proteins have been shown to induce TGF-β signaling and attenuate BMP signaling [15, 16]. CTDSP1 also stabilizes SNAIL and C-MYC proteins by dephosphorylating a key serine residue [17, 18]. Further, *CTDSP1/2/L* genes have all been found to contain an intronic microRNA that belongs to the miR-26 family. These miRNAs have been shown to act synergistically with the CTDSP1 and CTDSPL proteins to dephosphorylate, and thus activate, pRb and block the G1/S cell cycle transition [19]. CTDSP2 has also been shown to inhibit cell cycle progression independently by activating Ras and p21 [20].

Due to involvement in these pathways, it comes as no surprise that the CTDSP1/2/L and the miR-26 family have been implicated in tumorigenesis. *CTDSPL* has been characterized as a tumor suppressor gene that is frequently deleted or mutated in many major epithelial cancers such as lung, renal cell and breast carcinoma [19, 21–23]. Further, all 3 proteins are down-regulated in hepatocellular carcinoma cell lines [19]. Comparatively, little is known about CTDSPL2. It has been shown to play a role in erythroid differentiation and BMP signaling [24, 25]. However, CTDSPL2 has not been previously linked to tumorigenesis.

In this work, we characterize *CTDSPL2* as a novel gene involved in oncogenesis and further characterize the role of *CTDSPL*. Specifically, we investigate the function of viral induced truncations of both genes in cancer. Overexpression of *CTDSPL* and *CTDSPL2* leads to changes in expression of ribosomal genes and genes involved in cellular migration and metabolism. We show that overexpression of both *CTDSPL* and *CTDPSL2* causes accelerated cellular migration in primary cell culture. Interestingly, expression of *CTDSPL2*, but not *CTDSPL*, protects cells from apoptosis induced by oxidative stress, indicating that the two genes may not be redundant. Importantly, the truncated viral fusion transcripts of both *CTDSPL* and *CTDSPL2* promote immortalization when overexpressed in primary cell culture.

## RESULTS

### *CTDSPL* and *CTDSPL2* are common integration sites in ALV-induced B-cell lymphomas

High throughput sequencing was used to identify retroviral integration sites in ALV-induced B-cell lymphomas [10]. Integration sites that are overrepresented in the sequencing data, either because of clonal expansion or because the gene is a common integration site between tumors, were selected for and therefore believed to be important in tumorigenesis.

**Figure 1:**
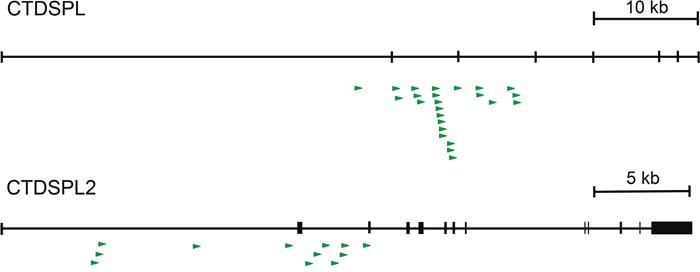
*CTDSPL* and *CTDSPL2* are common integration sites in ALV-induced B-cell lymphomas A schematic of retroviral integrations into both *CTDSPL* and *CTDSPL2*. Each integration is depicted as an arrow with the base of the arrow representing the site of integration. Direction of the arrow indicates the orientation of the retroviral integration with respect to transcription of the gene; all are in the sense orientation. There are 23 unique expanded integrations in *CTDSPL*, all of which fall upstream of exon 4. There are 13 unique expanded integrations in *CTDSPL2* upstream of exon 3.

*CTDSPL* and *CTDSPL2* were identified to be common integration sites previously [10]. In this study we have expanded our analysis and observed 23 unique clonally expanded integrations in *CTDSPL* in 12 tumors from 7 birds. All expanded integrations are in the same transcriptional orientation as *CTDSPL* and fall upstream of exon 4 (Figure 1; Supplementary Table 1). In addition, thirteen unique expanded integrations were detected in *CTDSPL2* in 7 tumors from 4 birds. All expanded integrations in the gene are in the same transcriptional orientation as *CTDSPL2* and fall upstream of exon 3 (Figure 1; Supplementary Table 1). No expanded integrations into either gene were observed in non-tumors. Interestingly, we did not observe integration into other CTDSP family members.

### Activation of *CTDSPL* and *CTDSPL2* are likely early events in tumorigenesis

**Figure 2:**
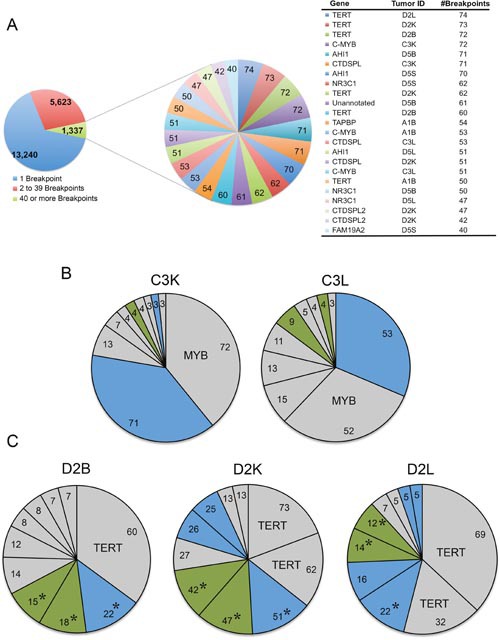
Viral integrations into *CTDSPL* and *CTDSPL2* are an early event in tumorigenesis **(A)** A composite pie chart of 11 tumors containing clonally expanded integrations in either *CTDSPL* or *CTDSPL2* is shown. In this tumor set, there were 14,879 unique integrations represented by 20,200 total breakpoints. Integrations having 40 or more breakpoints are depicted in a separate composite pie chart with individual integrations as a single “slice” of the pie chart, weighted by number of breakpoints. There are 23 integrations with 40 or more breakpoints. Of these top clonally expanded integrations, 3 are in *CTDSPL* and 2 are in *CTDSPL2*. **(B)** *CTDSPL* and *CTDSPL2* are dominant integrations in some individual tumors. The top 10 clonally expanded integrations are shown for representative tumors (C3L and C3K). In these cases *CTDSPL* and *CTDSPL2* are among the most dominant integrations. *CTDSPL* integrations are indicated in blue, *CTDSPL2* integrations are indicated in green. Top integrations are labeled. **(C)** Primary (bursa) and secondary (kidney and liver) tumors from the same bird (D2) have identical integrations in *CTDSPL* and *CTDSPL2*. *CTDSPL* integrations are indicated in blue, *CTDSPL2* integrations are indicated in green. The most dominant integrations are labeled. Identical integrations in each tumor are indicated with *. These integrations are clonally expanded in all cases, comprising a comparable proportion of the total tumor breakpoints. This suggests that these integrations occurred early in tumorigenesis, within the bursa and subsequently metastasized to these secondary sites.

A number of integration sites in the *CTDSPL* and *CTDSPL2* genes were found to be highly clonally expanded. Clonal expansion of a specific integration within a tumor was estimated via quantitation of sonication breakpoints as described previously [10]. The highest breakpoint integrations from tumors carrying *CTDSPL* and *CTDSPL2* integrations are shown in a composite pie chart in Figure 2A. In some individual tumors, these integrations were amongst the most dominant, expanded integrations (Figure 2B). This suggests that these integrations occurred early in tumorigenesis and were expanded as the tumor progressed.

Identical integration sites within both *CTDSPL* and *CTDSPL2* genes were identified in primary (bursal) and secondary (liver and kidney) tumors found in the same bird (Figure 2C). The presence of identical integration sites also indicates that these integrations likely occurred early in tumorigenesis prior to metastasis. The primary bursal tumor then metastasized to various locations including the liver, kidney and spleen, causing the clonal expansion of the integrated provirus in different secondary tumors. Interestingly, integrations in *CTDSPL* and *CTDSPL2* frequently occur in the same tumor. For instance, primary and secondary tumors in birds C3 and D2 have many of the most clonally expanded integrations in both genes (Supplementary Table 1).

### Viral integrations in *CTDSPL* and *CTDSPL2* drive the overexpression of genes

**Figure 3:**
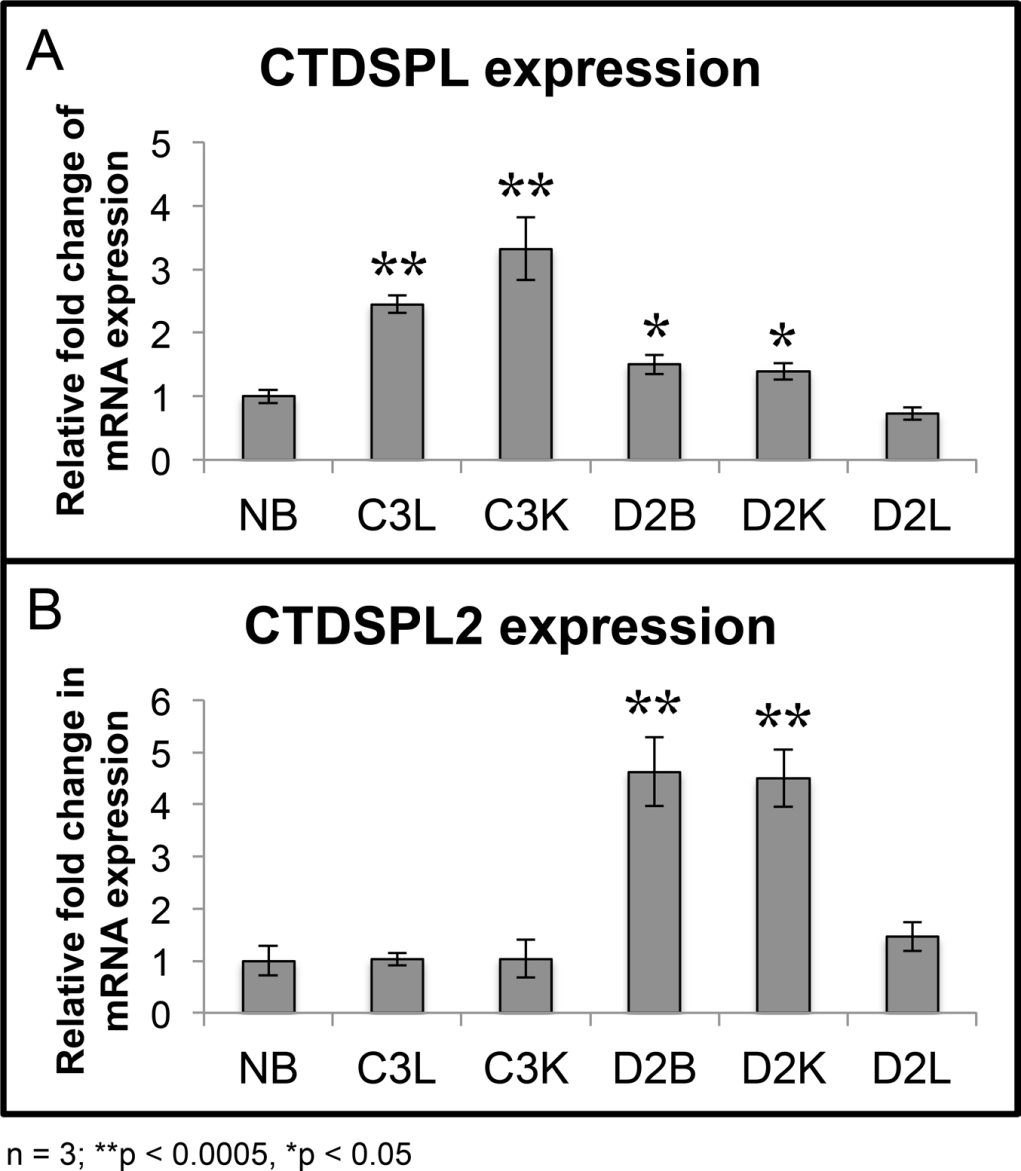
Tumors with expanded integrations in *CTDSPL* and *CTDSPL2* overexpress transcripts **(A)** qPCR was performed from tumor cDNA for either *CTDSPL* or *CTDSPL2* and normalized to a housekeeping gene, GAPDH. Fold change in mRNA expression is depicted relative to expression levels in normal bursa (NB). Tumors with the most highly expanded integrations in *CTDSPL* significantly overexpress *CTDSPL* mRNA by 3- to 3.5- fold (C3L and C3K). **(B)** Likewise, those tumors with the most expanded integrations in *CTDSPL2* also have a 4.5-fold increase in *CTDSPL2* mRNA expression (D2B and D2K).

Quantitative RT-PCR verified that relative to normal bursa, levels of both *CTDSPL* and *CTDSPL2* mRNA were elevated in the tumors with highly clonally expanded integrations in these genes (Figure 3A). For instance, C3L and C3K had a co-dominant integration in *CTDSPL* (Figure 2B) and expression of this gene was significantly elevated by approximately 2.5- to 3.5-fold respectively. Similarly, D2B and D2K had some of the most highly expanded integrations in *CTDSPL2* and we observed a corresponding 4.5 fold increase in expression (Figure 3B). It is interesting to note that tumors in D2 have clonally expanded integrations in both genes but only one of the genes is overexpressed.

### Integrations in *CTDSPL* and *CTDSPL2* generate truncated fusion transcripts

**Figure 4:**
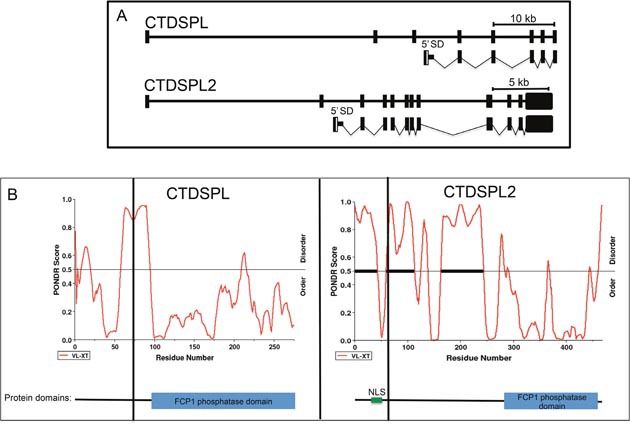
*CTDSPL* and *CTDSPL2* transcript truncations induced by viral integrations **(A)** Schematic of truncated transcripts expressed from integrations in *CTDSPL* and *CTDSPL2* as detected by RT-PCR. The promoter in the 5′ LTR of ALV drives expression of truncated transcripts. Transcripts contain the *gag* leader sequence spliced from the canonical splice donor site (5′ SD) into either exon 4 of *CTDSPL* or exon 3 of *CTDSPL2*. The ORF is not disrupted in either truncated transcript. **(B)** PONDR plots of CTDSPL and CTDSPL2. PONDR was used to predict intrinsically disordered regions of both CTDSPL and CTDSPL2 proteins [38]. PONDR VL-XT score is indicated by red line. Threshold for disorder is set at 0.5 and indicated by a horizontal line. Significant stretches of disorder are indicated by thick black horizontal lines. The N-terminal portion of CTDSPL2 is significantly more disordered than CTDSPL. Truncations induced by viral integrations are indicated by a vertical black line. In both CTDSPL and CTDSPL2, the truncations remove a portion of the predicted disordered region. For CTDSPL2, the truncation also removes a predicted nuclear localization signal (NLS). For reference the catalytic domain (blue box) and NLS (green) are shown in a schematic representation of CTDSPL and CTDSPL2 at the bottom of PONDR plots.

To determine the mechanism by which the viral integrations are disrupting *CTDSPL* and *CTDSPL2* expression, we performed RT-PCR to detect any potential viral fusion transcripts. We found that integrations in *CTDSPL* were driving the expression of a fusion transcript from the viral promoter with splicing occurring from the canonical splice donor site in *gag* to the splice acceptor site of exon 4 of the *CTDSPL* mRNA removing 77 amino acids from the N-terminus of the protein (Figure 4A). Integrations in *CTDSPL2* were driving expression of a fusion transcript from the viral promoter with splicing occurring from the canonical splice donor site in *gag* to the splice acceptor site of exon 3 of *CTDSPL2* removing 63 amino acids from the N-terminus of the protein (Figure 4A). In both cases, the viral start codon was in frame with the open reading frames and would add 6 amino acids of ALV *gag* at the N-terminus of the fusion protein. The truncation did not affect the catalytic phosphatase domain of either protein but did remove a portion of a predicted intrinsically disordered region of both proteins (Figure 4B). In the case of CTDPSL2, the truncation also removed a predicted nuclear localization signal (NLS; Figure 4B).

### CTDSPL and CTDSPL2 induce expression changes in genes implicated in cellular migration, translation, alternative splicing and oxidative phosphorylation

To better characterize the role of *CTDSPL* and *CTDSPL2* in ALV-induced B-cell lymphomas, we generated truncated transcripts in viral vectors to mimic those being expressed in tumors. Chick embryo fibroblasts (CEF) were infected with retroviral vectors (RCAS(A)) carrying either the truncated or full-length transcript of either *CTDSPL* or *CTDSPL2*. Transcripts were overexpressed approximately 100-fold relative to wild type CEF expression.

Both CTDSPL and CTDSPL2 are believed to act on the CTD of RNA polymerase II to regulate gene expression [11]. We reasoned that overexpression of these genes by viral integration may be affecting downstream gene expression. To identify changes in gene expression, RNA-seq analysis was performed on cells overexpressing truncated or full length *CTDSPL* or *CTDSPL2*. Cufflinks was used to detect genes differentially expressed in cells carrying a *CTDSPL* or *CTDSPL2* construct relative to cells infected with an empty retroviral construct.

**Figure 5:**
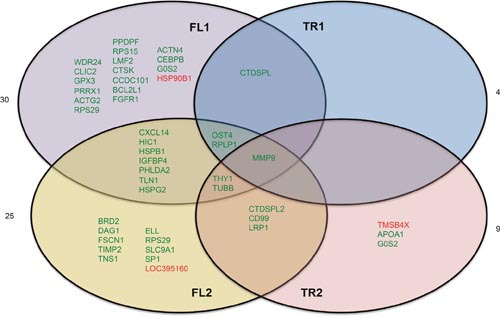
Genes differentially expressed by overexpression of *CTDSPL* or *CTDSPL2* full length or truncated transcripts RNA-seq analysis of CEF cells overexpressing truncated or full length *CTDSPL* or *CTDSPL2* revealed a number of significantly overexpressed genes. Venn diagram depicting deregulation of gene expression by overexpression of truncated or full length CTDSPL or CTDSPL2. Genes shown in green were significantly upregulated relative to cells infected with an empty viral vector. Genes shown in red were significantly downregulated. Fold change in expression for each gene is given in Supplementary Table 2.

We observed between 4 and 30 genes differentially expressed in each condition (Figure 5, Supplementary Table 2). There was very little overlap in differentially expressed genes between overexpression conditions. MMP9, or matrix metalloproteinase-9 is the only gene that was significantly deregulated by overexpression of all constructs. Cells expressing full length *CTDSPL* or *CTDSPL2* had the most similar changes in gene expression profiles with approximately 1/3 of the deregulated genes overlapping between the two conditions, suggesting that they may play partially redundant roles.

In order to determine differences in gene regulation induced by truncation of *CTDSPL* or *CTDSPL2* genes, we performed a GO analysis of genes differentially expressed between the full length and truncated form of both *CTDSPL* and *CTDSPL2* separately (Table 1; Supplementary Table 3). Differentially regulated genes between truncated and full length *CTDSPL* were enriched for genes involved in mitochondria, oxidative phosphorylation, alternative splicing and Sp1 targets. Mitochondrial genes as well as genes involved in oxidative phosphorylation were found to be upregulated in the cells expressing truncated *CTDSPL*. Sp1 target genes and genes involved in alternative splicing were found to be downregulated in cells expressing the truncated construct.

**Table 1:** Gene ontology (GO) analysis of genes differentially regulated by overexpression of truncated versus full length *CTDSPL* or *CTDSPL2*

GO term	P-value
***Enrichment in genes upregulated in TR1 vs. FL1***	
Metabolism	0.00000125
Oxidative phosphorylation	0.00000301
Ribosome	0.000927
Adherens/anchoring junctions	0.00471
Factor: E2F	0.00997
***Enrichment in genes downregulated in TR1 vs. FL1***	
Focal adhesion	6.47×10^−10^
Factor: ETF	0.00000002
Factor: Sp1	0.00000026
Factor: E2F-3	0.00000897
Cell migration	0.000286
Alternative splicing	0.0000654
***Enrichment in genes upregulated in TR2 vs. FL2***	
Ribosome	0.046
Cell adhesion	0.05
***Enrichment in genes downregulated in TR2 vs. FL2***	
Focal adhesion	0.0062
Ribsomal protein	0.0157

In both cases an enrichment of genes involved in cellular locomotion or focal adhesion, E2F targets and ribosomal genes were observed. There was no clear trend of upregulation or downregulation of the genes in these GO categories. There was little overlap in affected genes between full length and truncated constructs. Expression of truncated *CTDSPL* or *CTDSPL2* induced fewer changes in gene expression than either full-length construct indicating that the truncation may result in a partial loss of function.

### *CTDSPL* or *CTDSPL2* expression induces cell migration *in vitro*

**Figure 6:**
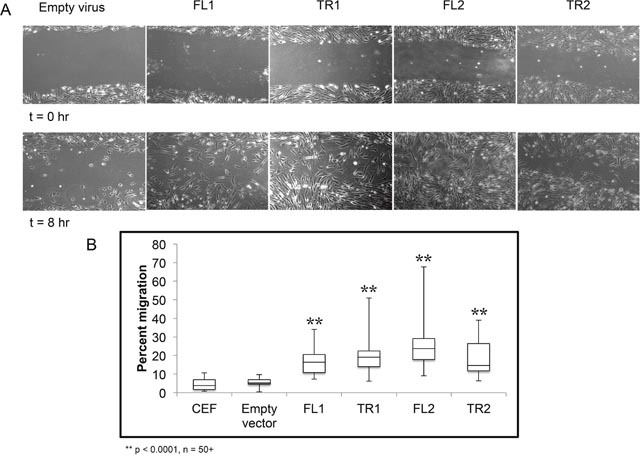
*CTDSPL* and *CTDSPL2* promote cellular migration in chick embryo fibroblasts **(A)** A scratch assay was performed to monitor cell migration. Representative images of scratches at time 0 and at 8 hours are shown. **(B)** Quantification of wound closure. On average, uninfected CEF and CEF infected with empty viral vector exhibit approximately 5% wound closure after 8 hours. Cells expressing truncated *CTDSPL2* (TR2) exhibited significantly faster cellular migration rates with 18% wound closure on average after 8 hours (p < 0.0001). Cells expressing full length *CTDSPL2* (FL2) had the fastest migration with 25% wound closure at the final time point (p < 0.0001). Cells overexpressing CTDSPL truncated and full-length (TR1, FL1) transcripts had intermediate phenotypes with approximately 15-20% migration.

Due to the enrichment of genes involved in migration, such as *MMP9*, we next looked at whether cells overexpressing full length or truncated *CTDSPL* or *CTDSPL2* had any differences in ability to migrate. To do this, we made use of a wound healing assay, or scratch assay, in which a confluent plate of CEF cells was scratched to disrupt the monolayer. At subsequent times after inflicting the “wound”, cells were imaged to visualize cell migration (Figure 6A). We observed that cells expressing either full length or a truncated *CTDSPL* or *CTDSPL2* transcript had a significantly higher rate of cell migration compared to an empty vector control.

Cells migrating into the wound were quantified, and percent wound closure was calculated (Figure 6B). *CTDSPL2* full-length overexpression had the largest effect with 25% wound closure compared to just 5% closure seen in the empty vector control (p < 0.0001). The truncated form of *CTDSPL2* had a more modest effect with 18% closure observed on average (p < 0.0001; Figure 6B). Cells expressing *CTDSPL* truncated and full-length transcripts had intermediate migration rates.

### *CTDSPL2* overexpression prevents apoptosis induced by oxidative stress

**Figure 7:**
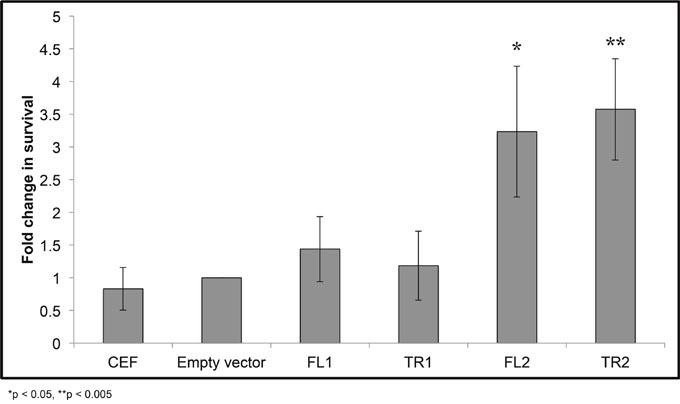
*CTDSPL2* protects cells from apoptosis *in vitro* Chick embryo fibroblasts were treated with hydrogen peroxide to induce apoptosis and survival was measured relative to cells infected with an empty viral vector. Expression of either full-length or truncated *CTDSPL* (FL1, TR1) provided no protection from apoptosis (p < 0.05). Cells expressing either truncated or full length *CTDSPL2* (TR2, FL2) showed an approximately 3-fold increase in cell survival (p< 0.05).

Promotion of cell migration by *CTDSPL* and *CTDSPL2* overexpression was observed when either truncated or full-length transcripts were expressed. Thus, this function does not explain why viral integrations that induce truncations were selected for in the tumors that we analyzed. Integrations in genes may also be selected for because they promote survival. To determine if integrations in *CTDSPL* and *CTDSPL2* are affecting survival, we induced apoptosis in cells expressing either full length or truncated *CTDSPL* or *CTDSPL2* by hydrogen peroxide treatment and measured cell death. Interestingly after 48 hours, cells expressing truncated or full length *CTDSPL2* had significantly higher survival rates than cells expressing *CTDSPL* or empty vector control. CEF cells expressing either form of *CTDSPL2* had approximately 3-fold higher survival than cells infected with an empty vector control (Figure 7).

### Overexpression of truncated viral fusion *CTDSPL* or *CTDSPL2* transcripts promotes immortalization of primary cells in culture

**Figure 8:**
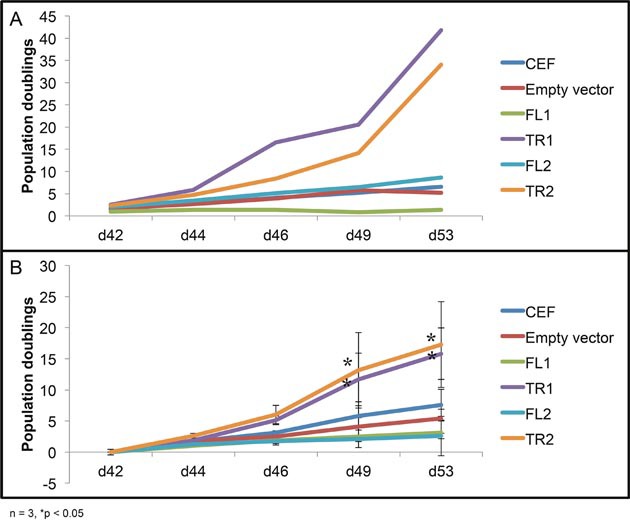
Overexpression of truncated *CTDSPL* and *CTDSPL2* promotes immortalization of primary cells in culture Proliferation data shown is from day 42 to day 53 after infection. **(A)** Representative growth curve of CEF cells infected with virus, expressing truncated or full length *CTDSPL* or *CTDSPL2*. **(B)** Average growth curve of three biological replicates. CEF cells as well as cells infected with empty virus stop proliferating at later time points. Cells overexpressing full length *CTDSPL* or *CTDSPL2* (FL1, FL2) proliferate less on average than uninfected CEFs. However, cells overexpressing truncated viral fusion transcripts of either *CTDSPL* or *CTDSPL2* (TR1, TR2) exhibit significantly higher proliferation at later time points indicating that they may be promoting immortalization.

The typical lifespan of primary chicken embryo fibroblasts in culture is approximately 30 days. After this point, proliferation of CEF cells as well as ALV-infected CEF cells decreases dramatically. Overexpression of either full length *CTDSPL* or *CTDSPL2* did not affect proliferation at later time points. In contrast, cells overexpressing the viral fusion transcripts of *CTDSPL* and *CTDSPL2* did not undergo senescence (Figure 8). These cells continued proliferating at the same rate that was observed at earlier time points (data not shown). This effect of the truncated products on immortalization is likely the reason integrations were selected for in our initial screen of ALV-induced B-cell lymphomas.

## DISCUSSION

In this report we have identified both *CTDSPL* and *CTDSPL2* as common integration sites in ALV-induced B-cell lymphomas. In addition to being common integration sites, a large number of integrations in these genes were clonally expanded, suggesting a role in tumorigenesis. Further evidence for a driving role in cancer is suggested by the presence of identical integrations in primary and secondary tumors within the same bird. This indicates that these integrations were likely an early event in the development of cancer within these birds.

We show that viral integrations in *CTDSPL* and *CTDSPL2* were driving the overexpression of a truncated transcript. Overexpression of *CTDSPL* and *CTDSPL2* caused changes in the expression of genes involved in cellular migration, most notably *MMP9*, which was upregulated by overexpression of all constructs. Correspondingly, we observed an increase in cellular migration rates in cells overexpressing truncated and full-length transcripts. This, in addition to the observation that integrations in *CTDSPL* and *CTDSPL2* occur in both primary and secondary tumors, suggests a potential role in promoting tumor metastasis. While CTDSPL2 is not well studied, it has been demonstrated to play a role in bone morphogenetic protein (BMP) signaling through dephosphorylation of Smad proteins [24]. This has been shown to strongly promote cell migration in hepatocellular carcinoma cell lines [26]. Further, inhibition of BMP signaling suppressed metastasis in mammary cancer [27]. This role of CTDSPL and CTDSPL2 in cellular migration agrees with previously published data that CTDSP1/2/L proteins promote migration through the activation of the SNAIL1 protein, a key regulator of migration [17]. The promotion of cellular migration appears to be a gain of function due to overexpression of the *CTDSPL* and *CTDSPL2* transcripts.

The overexpression of truncated viral fusion transcripts of both *CTDSPL* and *CTDSPL2* promotes immortalization of primary cells in culture. This is a feature unique to the truncated transcripts, as overexpression of full-length forms of both genes did not significantly improve proliferation rates at times past the normal lifespan of CEFs. We believe that this role in immortalization is likely the reason that integrations promoting the expression of truncated forms of both genes are selected for in ALV-induced B-cell lymphomas. This role in immortalization for CTDSPL and CTDPSL2 is interesting to note due to the co-occurrence of CTDSPL and CTDSPL2 integrations with integrations into TERT, which has previously been reported to promote immortalization [28].

*CTDSP1/2/L* are fairly well characterized genes that have been repeatedly shown to play partially redundant roles. CTDSPL2 seems to be fairly similar to the other members of the CTDSP family in many regards. Some functions are known to overlap, such as regulation of BMP signaling. Here we show that CTDSPL2 promotes metastasis similar to CTDSPL. These overlapping functions, in addition to the observation that despite integrations in both genes, only one is overexpressed in individual tumors, would suggest that CTDSPL and CTDSPL2 might be redundant. However, we observed that expression of *CTDSPL2*, and not *CTDSPL*, can protect cells from apoptosis induced by oxidative stress.

CTDSPL2 does have distinct features from the other members of the CTDSP family. For instance, *CTDSP1/2/L* genes have an intronic microRNA from the miR-26 family. No intronic microRNA has been reported in *CTDSPL2*. The CTDSPL2 protein, at 53 kDa, is significantly larger in size than CTDSP1/2/L proteins, which all weigh in at around 32 kDa on average. Each protein in the family contains a C-terminal phosphatase domain, but CTDSPL2 has significantly more N-terminal sequence of unknown function. The N-terminal region that is truncated in the viral fusion transcript is predicted to be intrinsically disordered (Figure 4B). Likewise, the truncated portion of CTDSPL is also predicted to be partially disordered. Given that the CTD of RNA polymerase II has been shown to associate with proteins with low-complexity domains, it is possible that these disordered regions are in part responsible for binding of CTDSPL and CTDSPL2 to the CTD. Thus, in the truncated transcript that is expressed in tumors, these proteins may not be able to associate with RNA polymerase II or other substrates.

Furthermore, unlike CTDSP1/2/L proteins, CTDSPL2 has not been previously reported to act on pRb, a main tumor suppressor target common to the other 3 members of the family. However, in our RNA-seq analysis, nearly 75% of the genes that were differentially expressed by 2-fold or more in cells overexpressing *CTDSPL2* were E2F target genes. E2F is a transcription factor targeted by pRb. When pRb is dephosphorylated and thus active, it binds E2F, keeping it inactive. Once pRb becomes phosphorylated in G1, it releases E2F allowing it to act on downstream effector genes causing the transition from G1 to S phase [30]. CTDSP1/2/L were shown to dephosphorylate and thus activate pRb [19]. Due to regulation of E2F target genes by CTDSPL2, it seems likely that this protein may also act as a phosphatase on pRb.

**Figure 9:**
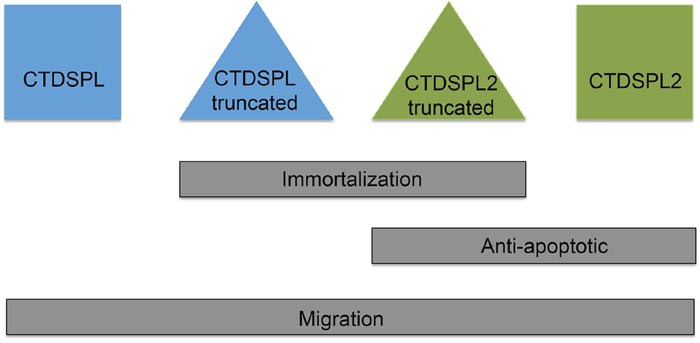
Summary of findings Overexpression of the truncated viral fusion transcripts of *CTDSPL* and *CTDSPL2* promote immortalization in primary cell culture. Expression of either full length or truncated *CTDSPL2* protected cells from apoptosis. Overexpression of all constructs caused a significant increase in cellular migration.

Our work suggests that *CTDSPL* and *CTDSPL2* play a role in cancer and seem to have pro-oncogenic characteristics (Figure 9). Expression of either of these genes promotes metastasis in cell culture and CTDSPL2 protects cells from apoptosis. Neither of these functions is affected by the viral truncation. We believe that the main reason integrations in *CTDSPL* and *CTDPSL2* were selected for in B-cell lymphomas is due to the role of the truncated transcripts in immortalization. We hypothesize that the gene truncations imposed by the viral integrations in tumors remove a region of the protein that is responsible for interaction with pRb. The truncated proteins would no longer be able to dephosphorylate pRb and would potentially lose their tumor suppressor function. Genes deregulated by expression of the truncated transcript were also enriched in downstream effectors and processes of the pRb pathway, such as E2F and Sp1 target genes [30]. This suggests that the truncated version of the proteins interacts with pRb differently causing a change in expression of downstream effectors of pRb. We hypothesize that the removal of a portion of a predicted intrinsically disordered region may inhibit these proteins from interacting with its normal protein-binding partners. For CTDSPL2, the truncation also removes a nuclear localization signal that may prevent the protein from reaching the nucleus. pRb has been shown to be a dominant effector of cellular senescence with inactivation of pRb delaying onset of cellular senescence [31, 32]. If the truncated CTDSPL and CTDSPL2 proteins can no longer activate pRb through dephosphorylation, then pRb may become phosphorylated and thus inactive, allowing for evasion of senescence as observed in our cell culture system.

## MATERIALS AND METHODS

### Tumor induction

The tumors in this study are rapid onset B-cell lymphomas induced by the subgroup A ALV virus, LR-9, or a variant thereof as described previously [10, 33]. Specifically, tumors in bird A1 and A8 were induced by delta LR-9, a variant with a 42 nt deletion in the *gag* gene that causes an increased frequency of read-through transcription [34]. Tumors in birds C2 and C3 were induced by an LR-9 variant with a point mutation, G919A. Tumors in birds D2, D3 and D5 were induced by wild type LR-9 virus. Tissue was collected from primary bursal tumors (B) or metastasized liver, kidney, or spleen (L, K, S) tumors.

### High throughput sequencing and analysis

Genomic DNA for ALV integration mapping libraries was collected using standard proteinase K digestion followed by phenol-chloroform extraction [6]. Libraries were prepared and analyzed using a custom pipeline as described previously [8]. Integrations were attributed to the nearest RefSeq gene. RNA-Seq libraries were prepared in duplicate using the TruSeq stranded mRNA library kit according to manufacturers directions and sequenced on the Illumina HiSeq platform. Differential gene expression between cells infected with a RCAS(A) viral construct carrying *CTDSPL* or *CTDSPL2* and cells carrying an empty vector control was determined using Cufflinks [35]. Genes with a 2-fold or greater difference in gene expression were considered for further analysis. Gene ontology (GO) analysis was performed using g:Profiler and DAVID [36, 37]. GO terms with a p-value of less than 0.05 after Bonferroni correction for multiple testing were considered significantly enriched above background.

### Characterization of truncated transcript and protein disorder prediction

RNA was extracted using RNA-Bee reagent (Tel-Test, Inc.). cDNA was prepared using Maxima H reverse transcriptase with an oligo(dT)_18_ primer (ThermoFisher Scientific). Fusion transcripts were detected by performing PCR with a forward primer in *gag* immediately before the viral splice donor (TCAAGCATGGAAGCCGTCATAAAG) and a reverse primer within the gene of interest (*CTDSPL*: TGAAAATGCAGTGCCTGTGC; *CTDSPL2*: CAGTA AGGTAGTTCGCGGGG).

Protein order was predicted using PONDR (Predictor of Naturally Disordered Regions) VL-XT [38]. Stretches of protein with 10 or more amino acids with a disorder prediction above the threshold 0.5 were considered potential disordered regions.

### Quantification of transcript abundance

qPCR to quantify transcript abundance was performed using PowerUp SYBR Green Mastermix (ThermoFisher Scientific) according to the manufacturer’s protocol on a BioRad C1000 thermocycler / CFX96 Real-Time System. Expression was measured using primers in either *CTDSPL* (CTACCTGTTGCAGAGTTTATGAAGC, TGAAAATGCAGTGCCTGTGC) or *CTDSPL2* (CCCCGCGAACTACCTTACTG, CAGCCTCAACA GCTTGTCCT). A housekeeping gene, *GAPDH*, was used as an internal reference [6]. qPCR was performed in triplicate and analyzed using the comparative C_t_ method (ΔΔCt)

### Cell culture, plasmid constructs, and viruses

Chicken embryonic fibroblasts (CEFs) were maintained at 39°C, 5% CO_2_ in 199 media supplemented with 1% chick serum, 1% calf serum, and 2% tryptose phosphate. Overexpression constructs were generated by cloning either full length or a truncated transcript into the RCAS(A) viral expression vector [39]. *CTDSPL* truncated construct begins at exon 4 (nt 281 from transcription start site in cDNA); *CTDSPL2* truncated construct begins at exon 3 (nt 348 from transcription start site in cDNA). Virus was generated via electroporation of constructs into CEFs with subsequent collection of the viral supernatant.

### Proliferation and apoptosis assay

Cells were seeded at 0.8 × 10^6^ cells in a 10 cm dish at time 0. To induce apoptosis, cells were treated with 50 μM H_2_O_2_ as described [40]. After 48 hours, cells were collected and counted using a BioRad automated cell counter (BioRad TC20) to determine change in cell survival relative to CEFs infected with empty viral vector. Population doublings were calculated from total live cell count at day 2 relative to day 0. Proliferation was then plotted relative to CEFs infected with empty viral vector as a control condition. Significance was assessed using an unpaired t-test.

### Scratch assay

A scratch assay was used to detect differences in cell migration as described previously [41]. Briefly, a 100% confluent plate of cells was scratched with a P200 tip at time 0. Closure of the scratch was monitored via light microscopy for 8 hours. Migration of cells into scratch was quantified using ImageJ [42].

## SUPPLEMENTARY MATERIALS TABLES









## Abbreviations

CTD: C-terminal domain
CTDSP: C-terminal domain small phosphatase
CTDSPL: C-terminal domain phosphatase-like
CTDSPL2: C-terminal domain phosphatase-like 2
CTDSP1/2/L: C-terminal domain phosphatase-1, C-terminal domain phosphatase-2 and C-terminal domain phosphatase-like
TR1: CTDSPL truncated form construct
FL1: CTDSPL full length construct
TR2: CTDSPL2 truncated construct
FL2: CTDSPL2 full length construct
ALV: avian leukosis virus
FCP1: (F-cell production 1)
LTR: long terminal repeat
RT-PCR: reverse transcription polymerase chain reaction
NLS: nuclear localization signal
CEF: chicken embryonic fibroblast
Sp1: specificity protein 1
pRb: retinoblastoma protein
MMP9: matrix metalloproteinase 9
