# Supplementary material for: Integration of ALV into CTDSPL and CTDSPL2 genes in B-cell lymphomas promotes cell immortalization, migration and survival
Source: Oncotarget. 2017 Jul 18;8(34):57302–15. doi: 10.18632/oncotarget.19328 (PMC5593642; doi:10.18632/oncotarget.19328)
Supplement: Supplementary file 1 [file oncotarget-08-57302-s001.pdf]

Integration of ALV into *CTDSPL* and *CTDSPL2* genes in B-cell lymphomas promotes cell immortalization, migration and survival

SUPPLEMENTARY MATERIALS

Supplementary Table 1: Genome coordinates, breakpoints and tumor information for integrations into CTDSPL and CTDSPL2

All clonally expanded integrations (2 or more breakpoints) detected in the screen are listed with tumor ID, number of breakpoints and genomic coordinates that correspond to the site of integration. CTDSPL coordinates are on chromosome 2; CTDSPL2 coordinates are on chromosome 10.

See Supplementary File 1

Supplementary Table 2: Cuffdiff results comparing gene expression in cells expressing either truncated or full length CTDSPL or CTDSPL2 relative to cells infected with an empty viral vector

Each tab corresponds to the Cufflinks output from a different construct relative to empty virus. FPKM values for each sample are listed as well as the $\log_2$(fold change) between the samples.

See Supplementary File 2

Supplementary Table 3: Detailed gene ontology (GO) information

GO terms that were enriched for genes up- or downregulated in cells overexpressing the truncated form of the gene versus full length. Genes that fall into each GO category are listed.

See Supplementary File 3
