# Supplementary material for: Integration of ALV into CTDSPL and CTDSPL2 genes in B-cell lymphomas promotes cell immortalization, migration and survival
Source: Oncotarget. 2017 Jul 18;8(34):57302–15. doi: 10.18632/oncotarget.19328 (PMC5593642; doi:10.18632/oncotarget.19328)
Supplement: Supplementary file 2 [file oncotarget-08-57302-s002.docx]

| **CTDSPL** | | |
| --- | --- | --- |
| Breakpoints | Tumor ID | Locus # |
| 71 | C3K | 4519921 |
| 53 | C3L | 4519921 |
| 51 | D2K | 4520683 |
| 26 | D2K | 4519498 |
| 25 | D2K | 4519227 |
| 24 | D2L | 4520683 |
| 22 | D2B | 4520683 |
| 18 | D5S | 4515471 |
| 16 | D2L | 4519227 |
| 14 | A1B | 4511874 |
| 13 | D5L | 4515471 |
| 12 | D2K | 4527016 |
| 10 | C2B | 4523438 |
| 6 | C2B | 4517279 |
| 5 | D2L | 4519498 |
| 5 | D2B | 4519498 |
| 5 | D2B | 4527016 |
| 4 | D2L | 4519620 |
| 4 | B6B | 4520674 |
| 4 | D2L | 4524658 |
| 4 | D2L | 4526898 |
| 4 | D2K | 4526898 |
| 4 | D2L | 4527016 |
| 3 | D2B | 4519344 |
| 3 | D2K | 4519620 |
| 3 | D2B | 4519675 |
| 3 | C3K | 4519923 |
| 2 | D5B | 4515471 |
| 2 | D9B | 4517448 |
| 2 | D5S | 4517790 |
| 2 | D2B | 4519227 |
| 2 | D2K | 4519827 |
| 2 | D2K | 4520864 |
| 2 | D2K | 4521356 |
| 2 | C3L | 4523454 |
| 2 | D2K | 4526408 |
| **CTDSPL2** | | |
| Breakpoints | Tumor ID | Locus # |
| 47 | D2K | 19283543 |
| 42 | D2K | 19283421 |
| 18 | D2B | 19283421 |
| 15 | D2B | 19283543 |
| 15 | D2L | 19283543 |
| 12 | D2L | 19283421 |
| 10 | D2K | 19296421 |
| 9 | D2K | 19282355 |
| 8 | C3L | 19284002 |
| 4 | D2L | 19282355 |
| 4 | C3L | 19285319 |
| 4 | C3K | 19296007 |
| 4 | D2B | 19296421 |
| 3 | D3K | 19286358 |
| 3 | D2L | 19296421 |
| 2 | D2B | 19282355 |
| 2 | D2B | 19284446 |
| 2 | D2K | 19284889 |
| 2 | C3K | 19285319 |
| 2 | D2K | 19291118 |
| 2 | C3L | 19296007 |
| 2 | D2B | 19296037 |
| 2 | A8B | 19310663 |
