# Supplementary material for: Integration of ALV into CTDSPL and CTDSPL2 genes in B-cell lymphomas promotes cell immortalization, migration and survival
Source: Oncotarget. 2017 Jul 18;8(34):57302–15. doi: 10.18632/oncotarget.19328 (PMC5593642; doi:10.18632/oncotarget.19328)
Supplement: Supplementary file 4 [file oncotarget-08-57302-s004.docx]

| **GO term** | **P-value** | **Genes** |
| --- | --- | --- |
| ***Enrichment in genes upregulated in TR1 vs. FL1*** | | |
| **Metabolism** | 0.00000125 | PSMB1, MGST1, MSMO1, OAT, ASNS, RPL31, NUP37, NDUFB2, PSMA6, SQLE, PPP2CB, PRKAG2, RPL34, NDUFA8, RPL5, AMD1 ,UQCR11, PAICS, DCTD, COX4I1, RAN, NDUFB5, HADH, LBR, RPL37, GSTO1, ATP5G3, CMPK1, HPGDS, RPL22L1, STARD4, CYB5A, IDH3A, ACAA2, UQCRFS1, SLC25A6, BPGM, RPL38, UQCRH, FAR1, RPL12, SDHD |
| **Oxidative phosphorylation** | 0.00000301 | NDUFB2, NDUFA8, UQCR11, ATP6V1E1, COX4I1, NDUFB5, ATP6V1G1, ATP5G3, UQCRFS1, UQCRH, SDHD |
| **Ribosome** | 0.000927 | MRPS35, RPL31, RPL34, MRPS14, RPL5, RPL37, RPL22L1, RPL38, RPL12, MRPS17 |
| **Adherens/anchoring junctions** | 0.00471 | RPL31, CHMP2B, RPL34, PRDX1, RPL5, PAICS, RAN, RRAS2, ACTR2, ITGAV, G3BP1, TWF1, CCT8, ANXA5, RPL38, RPL12 |
| **Factor: E2F** | 0.00997 | PSMB1, GLT8D1, MSMO1, MRPS35, OAT, RPL31, WDR1, SLC25A3, PREP, NDUFB2, TXN2, PSMA6, PGRMC1, RP2, SQLE, PPP2CB, PRKAG2, UFSP2, TMEM30A, USP4, PRDX1, STMN1, UCHL3, NDUFA8, UFM1, TBC1D15, SPRYD7, AMD1, UQCR11, PAICS, DCTD, LSM7, ATP6V1E1, COX4I1, CHMP1A, RAN, TPT1, SLC38A2, CDC73, MPHOSPH6, ATP6V1G1, ACTR2, ITGAV, HADH, NEDD1, TSPAN3, CDR2, LBR, RPL37, G3BP1, GSTO1, N6AMT2, TWF1, UCHL1, VMA21, CMPK1, MEMO1, HPGDS, ATP1A1, SLMAP, H2AFZ, ANXA5, STARD4, VDAC2, CYB5A, IDH3A, PHB, SNRPD1, CRK, ACAA2, UQCRFS1, SLC25A6, ARL6IP1, RNF139, RAB33B, BPGM, RPL38, UQCRH, AP3S1, MCFD2, ALDH1A3, YTHDF3, GMFB, FAR1, CAPZA2, TMSB4X, VDAC1, MRPS17 |
| ***Enrichment in genes downregulated in TR1 vs. FL1*** | | |
| **Focal adhesion** | 6.47x10^-10^ | CD99, ITGA3, PPP1R12A, PABPC1, TNS1, PPFIBP1, PTK7, LRP1, FLOT2, RAC1, TLN1, GIT2, HSPG2, THY1, ZYX, RPL8, ARF6, ADAM9, YES1, EVL, TGM2, MARCKS |
| **Factor: ETF** | 0.00000002 | CD99, ITGA3, LAMP2, MAP2K3, TIMP2, VCAN, FAM214A, MRTO4, PPP1R12A, HDAC7, WAPAL, NAV3, MEF2A, PABPC1, NDE1, PTGS2, FGFR1, SMARCA2, TDRD3, AK6, CCNK, FKBP5, KDELR3, CHMP4B, NDFIP2, TSC22D1, FAM173A, SF3A2, MEOX2, RSRC2, ELK3, CPSF6, RWDD1, PTK7, IK, CISH, NCL, SPTBN1, HPCAL1, PRRX1, B4GALT2, PRDX6, EGR1, CLU, LRP1, G0S2, STAU1, PPDPF, WDR24, WDR44, FLOT2, MYH10, SRRM1, BHLHE40, PUM1, FADS2, RAC1, TLN1, ETV6, GIT2, MFAP1, PARN, IGFBP4, COL6A1, COL6A2, HSPG2, RIT1, SNRNP200, METTL21A, CTDSPL, ADPRH, CCNA2, SFRP2, CXCL14, ADD3, ADAM33, HNRNPDL, CFDP1, THY1, ELMO1, EIF5B, RNF166, ZYX, RPL8, PPAP2B, ZNF326, PM20D1, RPRD2, CDCA7L, ARF6, TAF3, MAPRE2, LINGO1, SIX2, PDGFD, CANT1, HDAC3, PTPN2, YES1, BASP1, PTRF, PHLDA2, PRKAG1, COL18A1, EWSR1, TOP1MT, PDE4B, SP1, COL4A1, NOC2L, H1F0, SRC, SPG7, MAFK, BRD2, GPX3, MARCKS |
| **Factor: Sp1** | 0.00000026 | CD99, ITGA3, LAMP2, MAP2K3, TIMP2, VCAN, FAM214A, PPP1R12A, HDAC7, NAV3, MEF2A, PABPC1, NDE1, FGFR1, TNS1, SMARCA2, AK6, CCNK, FKBP5, KDELR3, CHMP4B, NDFIP2, TSC22D1, FAM173A, SF3A2, CBLL1, PPFIBP1, RSRC2, ELK3, CPSF6, PTK7, IK, CISH, NCL, SPTBN1, HPCAL1, PRRX1, B4GALT2, PRDX6, DUSP1, EGR1, CLU, CDK2, LRP1, STAU1, PPDPF, WDR44, SERPINF1, FLOT2, NES, MYH10, SRRM1, BHLHE40, FADS2, RAC1, TLN1, ETV6, MFAP1, MFGE8, PARN, IGFBP4, COL6A1, COL6A2, HSPG2, RGL1, RIT1, SNRNP200, METTL21A, CTDSPL, ADPRH, CCNA2, SFRP2, CXCL14, ADD3, ADAM33, HNRNPDL, CFDP1, THY1, EIF5B, RNF166, RUNX1, ZYX, RPL8, PPAP2B, ZNF326, PM20D1, RPRD2, ARF6, MAPRE2, ADAM9, UGP2, LINGO1, SIX2, PDGFD, CANT1, BCL2L1, HDAC3, PTPN2, YES1, CCDC101, BASP1, PTRF, PHLDA2, PRKAG1, COL18A1, EWSR1, TOP1MT, PDE4B, SP1, COL4A1, NOC2L, H1F0, SUPT5H, SRC, SPG7, MAFK, TGM2, BRD2, GPX3, MARCKS |
| **Factor: E2F-3** | 0.00000897 | CD99, ITGA3, LAMP2, MAP2K3, TIMP2, VCAN, FAM214A, HDAC7, WAPAL, NAV3, MEF2A, PABPC1, NDE1, FGFR1, SMARCA2, TDRD3, AK6, CCNK, FKBP5, KDELR3, CHMP4B, TSC22D1, FAM173A, SF3A2, PPFIBP1, ELK3, CPSF6, PTK7, IK, CISH, NCL, SPTBN1, HPCAL1, PRRX1, B4GALT2, PRDX6, DUSP1, EGR1, CLU, CDK2, LRP1, STAU1, PPDPF, WDR44, SERPINF1, FLOT2, NES, MYH10, SRRM1, BHLHE40, FADS2, RAC1, TLN1, ETV6, MFAP1, MFGE8, PARN, IGFBP4, COL6A1, COL6A2, HSPG2, RGL1, RIT1, METTL21A, CCNA2, ADD3, ADAM33, HNRNPDL, CFDP1, THY1, ELMO1, RNF166, RUNX1, ZYX, RPL8, ZNF326, PM20D1, RPRD2, CDCA7L, ARF6, TAF3, MAPRE2, ADAM9, UGP2, LINGO1, SIX2, CANT1, BCL2L1, HDAC3, PTPN2, YES1, CCDC101, BASP1, PTRF, NQO1, PHLDA2, PRKAG1, COL18A1, EWSR1, TOP1MT, SP1, COL4A1, NOC2L, H1F0, SRC, SPG7, MAFK, TGM2, BRD2, GPX3, MARCKS |
| **Cell migration** | 0.000286 | ITGA3, HDAC7, NDE1, PTGS2, FGFR1, TNS1, CBLL1, MEOX2, ARHGDIB, PTK7, LRP1, SERPINF1, MYH10, RAC1, SFRP2, CXCL14, THY1, ELMO1, PPAP2B, ADAM9, SIX2, PDGFD, YES1, PHLDA2, COL18A1, PDE4B, EVL, SRC |
| **Alternative splicing** | 0.0000654 | TSC22D1, LAMP2, LRP1, CTDSPL, COL6A2, PPP1R12A, PUM1, VCAN, BCL2L1, SRC |
| ***Enrichment in genes upregulated in TR2 vs. FL2*** | | |
| **Ribosome** | 0.046 | RPS2, MRPL33, RPS3 |
| **Cell adhesion** | 0.05 | RPS2, MRPL33, RPS3 |
| ***Enrichment in genes downregulated in TR2 vs. FL2*** | | |
| **Focal adhesion** | 0.0062 | CAV1, RPS29, RPLP1, DAG1, CAPN2 |
| **Ribsomal protein** | 0.0157 | MRPS26, RPLP1, MRPL20 |
